# Just Like a Woman: Gender Role Stereotypes in Forensic Psychiatry

**DOI:** 10.3389/fpsyt.2022.840837

**Published:** 2022-04-04

**Authors:** Saima Ali, Gwen Adshead

**Affiliations:** West London NHS Trust, Southall, United Kingdom

**Keywords:** gender, stereotype, forensic, psychiatry, mental health

## Abstract

The relationship between violence, gender and mental health is a complex one which is yet to be fully understood. Gender role stereotypes are social constructs that can powerfully influence and regulate human behaviour, including violence; and so it is likely that they also influence the nexus of violence management and mental health which is at the core of forensic psychiatry. In this article, we examine how gender role stereotypes might influence the practice of forensic psychiatry: specifically, in relation to women as violent offenders, as patients in secure psychiatric care and as clinicians working in forensic settings. We identify areas of development in women’s forensic mental health services, and examine whether patriarchal influences and gender role stereotypes may have inadvertently impacted upon these changes. We also consider whether these changes may maintain pre-existing barriers to treatment for both men and women.

## Introduction

In this paper, we will explore how gender role stereotypes and expectations might influence practice in forensic mental health settings. Specifically, we suggest that these stereotypes may operate both implicitly and explicitly in ways that are harmful to women’s mental health, and consider how such an operation may be especially problematic for women involved in forensic services. We will further suggest that the operation of gender role stereotypes in forensic mental health services is not just a matter for female patients in forensic services but also extends to female professionals in secure hospital settings and within the legal system.

We begin with an overview of gender as a social construct and consider how gender role stereotypes impact upon how psychological distress is communicated by men and women. We then turn to the intersection of gender role expectations and their influence on antisocial behaviour and states of mind, with reference to the role of the forensic psychiatrist in assessing the functional link between violence risk and diagnosis. We discuss some responses of the legal and criminal justice system, as well as the media, to female violence, and conclude with some consideration of the gendered experience of female professionals working in forensic settings.

We have to declare a major caveat about the scope and depth of what is discussed. The academic domains of gender, crime, violence, and mental health are all vast, and any kind of detailed systematic review of how they intersect would lead to a book length publication, with multiple volumes. We therefore do not claim to provide a detailed or definitive analysis of all arguments in this paper. Our intention is to raise awareness of these complex constructs by pointing to related literature that has posed similar questions. We are aware that by focussing on Anglophone countries, we have not been able to offer any comment on the intersection between ethnicity, culture and religious beliefs with gender-based prejudice and related concerns about legal and mental health practice; especially in those countries where forensic mental health services are still emerging. These equally important and relevant concepts deserve study in their own right. In this brief overview, our aim is to generate discussion and reflection about gender role bias within forensic mental health services which can enhance awareness and potentially improve practice.

## Gender and Gender Role Expectations

The concept of gender is complicated and the term can be used in different senses in ways that cause confusion. Stock [(1) p. 38] describes four different senses in which the term is used: first, a general term for the division of the sexes; second, referring to social stereotyping about sexes; third, referring to projections of ideas about masculinity and femininity onto men and women; and fourth, where gender is shorthand for “gender identity.” Given these different senses in which the word is used, there is scope for confusion and disagreement. For our purposes, and because our discussion is in the context of health care, we will use the WHO definition of gender which is defined as “*the characteristics of males and females that are socially constructed, and includes norms, behaviours and roles associated with being of either sex*” (2).

Traditional accounts of gender typically offer a binary divide of “masculinity” and “femininity” in terms of what the two sexes are “like” psychologically and socially. Early historical accounts of gender were thought to reflect natural expressions of an individual’s sex chromosomes, which were “normal” because “natural.” However, this conception of the relationship between sex and gender has been regularly challenged as superficial by historians, feminists, biologists and social anthropologists who have studied male and female roles and relationships in different societies across time and using different methods. Many commentators have observed that gender role beliefs and expectations become social stereotypes that serve as regulators of social relationships in human groups, especially in relation to power and control over property, social and reproductive status (3–5). Carlen (6) asserted that women were exploited in “gender deals” that kept women in domestic roles in exchange for love and financial support from their husbands, as well as respectability. Butler’s gender performativity theory views gender as a set of learned behaviours, akin to a “performance,” in order to fit into social constructed notions of “male” and “female” (7).

Rigid definitions of “masculinity” and “femininity” lead to the development of gender role stereotypes that can have harmful effects for both men and women in different life domains; including work, relationships, social status, and health. For example, a definition of femininity that emphasises passivity and inability to act under pressure can result in a social expectation that women cannot lead or take important decisions. If women are then prevented from taking leadership or active roles on the basis of this stereotype, then they will by default be unable to prove it false, and their absence from these roles is then taken as evidence of their “natural” passivity. Similarly, if gender role expectations of masculinity emphasise strength, competition and lack of nurturing capacity, then young men and boys are likely to act into those roles and exclude themselves from nurturing roles; which then accentuate the notion that it is a naturally feminine task.

One pervasive gender role stereotype in relation to women’s social roles assume that it is “normal” and natural for women to provide care for others, so they will “naturally” dominate in the care-giving professions (8). However, there is nothing “natural” about remunerating care work at a much lower rate than other similar forms of manual labour carried out by men. Men are paid at higher levels than women, in both manual and professional settings, when they are doing the same job; the current gender pay gap in the United Kingdom is 7.9% (9). Such inequality in pay for the same role suggests that work by women is rated as less valuable than work by men.

Research methods themselves may be affected by gender role stereotyping, in terms of methodology, sampling bias and the theme of the research itself. Research into any kind of sex difference often starts from the assumption that male data is the “norm” and female data is a variant or deviant from the norm. There is evidence of this kind of bias in relation to the study of pathology in physical health. For example, the “textbook” description of symptoms of myocardial infarction have been those which are regularly reported by males and thus typically true for them. However, research suggests that this description is not typical for females who experience different symptoms with the same disease (10). Similarly, males and females may experience pain or metabolise drugs differently; but if the “male” profile is deemed to be the norm, the female sex and gender role differences may not be identified (see (11)). In this way, biased gender role stereotyping can impact negatively on treatment and management of a range of health conditions for both sexes (12). Such bias may have been a particular issue in relation to women’s capacity for cruelty and antisocial behaviour by positing male violence as essentially “normal” and women’s violence and cruelty as evidence of mental disorder [for a general discussion see (13)].

Gender role stereotypes can also influence how men and women express psychological distress, and how they manage painful emotions which affect their well-being (such as sadness, fear, and anger). Gender role expectations for men which emphasise strength, dominance over others and invulnerability may encourage men to externalise their distress in terms of bodily action; and may contribute to increased rates of suicide and homicide in males (14, 15). Further, this gender role expectation generates an opposing one for women i.e., the belief that it is normal for women to be able to easily articulate distress to others. However, both adolescent boys and girls may struggle to communicate distress verbally and may use their bodies as vectors of pain: boys describe more problems related to anger, engage in higher-risk behaviours and commit suicide more frequently than girls (16), whereas girls experience suicidal ideation but express distress by harming their own bodies or developing eating disorders.

These trends continue into adulthood, as a greater proportion of women report anxiety, hopelessness and helplessness whilst men tend to engage in antisocial behaviours that are problematic for others (17). Historically, when female patients presented with “masculine” symptoms (such as alcohol dependence or antisocial behaviours), they were often viewed as suffering more severe mental disturbance and the same was true for males presenting with “feminine” symptoms such as depression (18). Gender role expectations have also played a part historically in how women’s mental health was assumed to be vulnerable in pregnancy and menstruation, and those women who did not appear to enjoy motherhood were more likely to be seen as mentally unwell (19).

More recently, there has been increased concern about those individuals for whom gender role stereotyping does not “fit” their sense of lived identity, which can cause mental distress. Although previously such gender “dysphoria” was deemed to be a mental health condition which required psychosocial treatments, the World Health Organisation (WHO) now state that such distress does not constitute a mental health problem (20).

We are discussing here the harmful impact of gender role expectations, beliefs and stereotypes at the level of large-scale communities and groups. Within those groups there will be individual exceptions and variations, and on an individual basis, there may be many men and women who feel comfortable with the gender role in which they have grown up and been socialised. However, they may not be aware of the limitations of the gender role in which they have been raised, nor the impact on their relationships with others, because of the cultural beliefs which structure their society. For example, feminist academics such as Gilligan and Richards (21) have argued that gender role stereotypes about male and female “norms” underpin a wider and more implicitly entrenched system of social beliefs, (usually described as “patriarchal”) which assume a dominant role of men in society in terms of decision makers and controllers of those who are more vulnerable. Such patriarchal systems are harmful to both men and women, at an individual and a social level, because patriarchal thinking views vulnerability and neediness as shameful and relationships as solely transactions regulated by strength and domination. Such an analysis leads to gender inequalities which may have particular implications for how violence is understood in patriarchal societies and forensic services [e.g., (22)]. Patriarchal societies tend to have higher rates of homicide and suicide, especially if the political systems support patriarchal values; the effect is noticeable when comparing homicide and suicide rates in the United States with those in Europe (23).

## Crime, Gender and Violence Risk

Within criminology, debates about gender and crime began in the 1970s and 1980s (24). Early debates in criminology focused on the question of whether data from studies of male offending could be generalised to female offenders; or whether female criminality (especially violence) might be specific to women’s role in society; or whether female offenders were deviant compared to non-offending women (24, 25).

Human violence is not homogenous and is arguably best understood as a transaction between individuals within a particular social context (26). (In this context, we are excluding violence in terms of organised wars and conflicts that have social support and endorsement). It would seem reasonable to assume that gender role expectations and stereotypes might be relevant to the analysis of human violence, especially when it is known that at least 80% of violence perpetrators are male; a figure that appears to be the same across countries and cultures (27). However, although most violence perpetrators are male, most men are not violent and in most community populations, the denominator of non-violent males is large. Overall, violence is an uncommon way for people to break the criminal law, and rates of violence have been dropping in most social democratic societies over the last 4 decades. (This is even true of the United States, despite the marked elevation of their homicide rate by gun ownership.)

Some early theoretical models of violence do not mention gender at all. An early and influential paper by Bronfenbrenner (28) described an ecological model of risk factors for violence arising from both the macro-culture of the society and the micro-culture of the individual. Macro risk factors for violence include peer pressure, effects of deprivation and exclusion, and the creation of deviant/dissenting sub-cultural groups. Micro risk factors include neurophysiological and psychological risk factors, such as attitudes to rule breaking and violence in families and communities, and belief/value systems that are unempathic or antisocial. There is no mention of gender, either as a macro- or micro-level risk factor; despite the model being an attempt to reduce tension between criminological models and psychological models of risk for crime.

If gender role were included as a risk factor for violence, then it would be tempting to see masculinity as a major risk factor. For example, Lantz (29) quotes one writer who describes violence as “a resource for demonstrating masculinity,” which might suggest that violent women are unusually “masculine.” At least one risk assessment tool rates being female as a protective factor against violence [e.g., the VRAG, (30)], which makes the tool hard to use with female violence perpetrators. There seems to be strong support for the view that, while it is usually illegal and unwelcome, male violence is essentially normal, as are the motivations for male violence. For example, those who support an evolutionary perspective argue that males may be motivated to engage in violence in order to protect their reproductive status and authority over other male rivals (31). Anger, protection, social recognition, perceived positive outcomes and pleasure have all been posited as motivations for male violence (32). Motivations for female violence are often assumed to be different to male violence without much evidence to support this assumption: even although absolute rates of violence are far lower in women than in men, the motivations for female violence appear to be similar (13, 33, 34).

Such gender-based assumptions are mirrored in relation to the sex of victims of violence. Gender role stereotypes of women often include a narrative of victimisation experience; and yet, in terms of fatal violence at an international level, overall, men are still more likely to be murdered than females. However, context is crucial to make sense of this: males are more likely to be murdered in countries where the homicide rate is linked closely to the drugs trade, whereas women are more likely to be victims in countries where drug related crime is low, and relational violence then is proportionally more common (35). Another example of how victimisation rates for both sexes are similar in one way but different in another was reported in a large-scale epidemiological study of 34,000 people (36). This study found that heterosexual men and women report similar levels of violent victimisation, but the nature and context of that violent victimisation is very different for men and women. Adult males reported higher levels of assaults by strangers with weapons, and non-partner violence; but women report higher prevalence of intimate partner violence (IPV) and sexual assault, both in childhood and adulthood. This study also found that “sexual minority” (*sic*) men and women were at increased risk of victimisation, suggesting that people who violate gender role expectations may face increased risk of attack.

Violence against women may be under-reported because it occurs in the domestic sphere, and there is evidence that violence against women is an international public health concern (37). Although most IPV perpetrators are male, there is evidence that females can also be perpetrators of violence of IPV, although this issue is less well researched. A study by Williams et al. (38) found that (like their male counterparts), female IPV perpetrators typically begin with emotional abuse of partners and then progress to physical and sexual IPV. In the context of discussion of women’s motivations for violence, it is often postulated that IPV by women is motivated by fear and the need for self-defence. However, Swan et al. (39) studied women serving sentences in a federal prison in Canada; and found that of those who had a history of IPV, 64% had initiated the violence in at least one incident. Stewart et al. (40) studied the reported motives for violence in female IPV perpetrators and reported that self defence or defence of children were the *least* frequently coded motive. In a study of Saudi Arabian women who reported carrying out IPV, participants described using violence as a means of expressing frustration about patriarchal practices and wanting freedom from oppression (41). Finally, a systematic review of the literature on female IPV perpetrators’ motives for violence identified anger in response to a felt inability to get their partner’s attention: not dissimilar from male motivations described above (42).

These studies have important implications for interventions for women who commit IPV, who may need programmes that are both similar and different to their male counterparts. The difference in psychological treatment needs between male and female IPV perpetrators remains an area in need of further exploration (43). While male IPV offenders in prison or on probation may have access to offence-specific interventions which look at gender role stereotypes and prejudices, female IPV perpetrators may be offered (a) programmes designed for males, where females are always victims, (b) general violence reduction programmes which do not look at the relational context, or (c) programmes that focus on women’s experience of victimisation and not their capacity for anger and revenge.

It is rare for women to commit acts of fatal or serious violence, but when they do, the violence can resemble male violence in terms of attacks on vulnerable victims. For example, women are frequently responsible for the deaths of their dependent children (44), just as men are responsible for fatal and non-fatal violence toward dependent partners. Rates of female violence appear to be increasing over time, and their violence risk is influenced by anger, hostility and substance misuse, just like their male counterparts. Intriguingly, both male and female violence perpetrators report similarly high levels of childhood adversity (45–48); suggesting that early and prolonged exposure to fear may be a risk factor for later violence.

Why the absolute numbers of violence perpetrators should be so different between the sexes remains an open question, and the answer is likely to involve an interaction of individual and social factors. Of those social factors, it seems reasonable to hypothesise that that gender role stereotypes play a role; whether it is in constructing normative accounts of masculinity in which violence is acceptable or accounts of femininity are based on victimisation. Some criminologists have argued that within masculinity, there exists a toxic variant which denigrates and degrades vulnerability in others in ways which increase the risk of violent attacks. Conversely, it may be that traditional gender role stereotypes of femininity are protective for women because they encourage social bonding and discourage the kind of social isolation that is known to be a risk factor for violence and poor mental health.

## Forensic Psychiatry, Mental Disorder and Violence Risk

Forensic psychiatry as a profession grew out of two observations; first, that some people who are violent are clearly mentally unwell at the material time, and second, that significant proportions of serving prisoners have mental health problems that require management and treatment. Forensic psychiatrists in Europe, Canada, Australia and New Zealand both assess and treat violence perpetrators with mental disorders (either in prison or secure services) and they also provide expert testimony on these issues. In the United States, forensic psychiatrists generally only provide expert testimony although clinical forensic services are growing. Similar services in non-western countries such as those of Sub-Saharan Africa and South Asia have been neglected and remain in early stages of development [e.g., (49, 50)]. However, whilst general psychiatric beds appear to be in decline, numbers of forensic mental health patients are rising internationally (51).

Forensic psychiatrists typically analyse, formulate and manage any potential functional link between mental disorders and violence. They offer assessments on this issue, and based on that formulation, may also offer care to people who are serving sentences for violence in prison, and who need psychiatric help. Although the treatment offered is primarily directed toward improving mental health, in practice, forensic psychiatrists also seek to help their patients reduce their risk of violent recidivism in the future; and violence risk management is a key role for forensic psychiatrists.

Forensic psychiatry has emerged out of general psychiatry, which in turn developed from a traditional medical model of mind and disorder. Since the 1990s, risk factors for violence and antisocial behaviour have been increasingly studied at the level of the individual, using bioscientific methods [e.g., (15, 27)]. Studies of the link between mental disorder and violence have found that mental disorder can increase violence risk, especially those conditions that cause intense paranoia and the sense that one’s control of thoughts is being over-ridden (52). Both antisocial personality disorder (ASPD) and substance misuse are associated with increased violence risk, although substance misuse probably has the greatest effect (53, 54). Other kinds of personality disorder are also known to increase risk in conjunction with ASPD, such as Narcissistic Personality Disorder (NPD) and Emotionally unstable personality disorder (EUPD; also known as borderline personality disorder or BPD). However, many sociological risk factors for violence are stronger than mental disorder (such as youth, poverty, substance misuse, and exposure to childhood adversity) and may carry greater predictive weight.

In forensic services, there are noticeable differences in the ways that diagnoses are made. ASPD is a diagnosis which is associated with both criminal offending and increased violence risk (55). It is also a diagnosis which is made more commonly in males, whereas EUPD is a diagnosis made more commonly in females (51). These diagnostic differences may reflect real differences in personality disorder presentation between the sexes, but may also reflect gender role stereotypes about criminal deviance. There may be a reluctance among clinicians to diagnose ASPD in women offenders, and Hodgins (56) highlights that most female aggressive and antisocial behaviour does not lead to prosecution. The same diagnostic reluctance may persist even in those women who have substantial criminal records (which is a diagnostic criterion for ASPD), and also to believe that male offenders may meet criteria for EUPD. In this context, it is noteworthy that the combination of ASPD and EUPD is common in violence perpetrators and may be associated with increased risk to self and others (57). It is also known that EUPD is associated with emotional dysregulation which mediate the risk of high levels of interpersonal conflict, which in turn leads to an increased risk of intimate partner violence (IPV). If EUPD goes largely unrecognised and untreated in the male population, then men with EUPD will be at increased risk of IPV while being deprived of evidence-based therapies for EUPD that might reduce both symptoms and risk.

Psychopathy is a disorder of personality which is known to be associated with an increased risk of violence. Studies of psychopathy in women over the last three decades suggest that gender role stereotypes influence how psychopathy is diagnosed in women (58–60). For example, it has been argued that sadistic and cruel attitudes (which exist in both sexes) are expressed differently by gender; so that women express their sadism in verbal, not physical ways; such as gossiping, excluding others from social groups, and criticising others (61, 62). Logan (63) suggests that, in comparison to similar males, females with psychopathic traits typically undermine the self-esteem and emotional wellbeing of their victims. But it might also be argued that verbal sadism is qualitatively different from physical sadism in terms of causing injury or death; to the point that apparent similarity may be meaningless. Further, the image of the gossiping, critical woman is another stereotype which may do little to help understand women’s capacity for cruelty and the extent to which this is essentially different from male cruelty. It may also distort assessments of violence risk in women if verbal cruelty is included; Skeem et al. (64, 65) suggested that female capacity for violence is underestimated by clinicians, particularly when they suffer from psychiatric disorders.

It has been suggested that for women, mental illness is a more important risk factor for violence than for males. For example, Hodgins (66) estimated that women with mental illness were 27 times more likely to be registered for a violent crime than those women without. However, what is puzzling about such data is that one might then expect rates of violent crime to be higher in women given that mental illness rates in women have been repeatedly reported as both high, and higher than in males (67, 68). Similarly, if mental illness were a risk factor for violence by women, then one might expect psychosis and other Axis 1 diagnoses to be frequently made in inpatient forensic services, but this is not the case. In inpatient forensic services for women, EUPD is the commonest diagnosis (69), but psychosis is by far the commonest diagnosis in male forensic inpatients (70). This difference in diagnosis may indicate that women’s violence is attributed more commonly to their personality disorder than mental illness, and is differently formulated compared to male patients.

Although women with mental illness appear to have higher rates of violence than women in the general population (52, 71), this may reflect a general underreporting of violence by women, especially if victims of female violence are children or family members, and if injuries may not be severe enough to warrant medical attention (72, 73). Women with mental illness may be better able than men to seek care and treatment. Mental illness is often used to explain female violence to children in a way which is not applied to males who attack children (74), including fatal violence. In the criminal courts, lawyers may seek to present their female clients as mentally ill, in order to make them seem both “normal” and sympathetic (75).

It is possible that forensic psychiatrists who evaluate women for criminal trials are influenced by gender role stereotypes that portray women who violate social roles as mentally ill. Psychiatrists may be invited to provide formulations that support legal strategies that depict a female defendant as a victim not a perpetrator, in terms of past trauma and a mental illness diagnosis. In homicide cases, the defence may seek to argue that the defendant was a victim of violence and coercive control and portray the deceased as cruel, a bully, or coercive and controlling. Such a defence is rarely successful and most women who kill their partners then change their defence to diminished responsibility on the grounds that they were suffering from a mental illness (often some form of PTSD due to being victimised by the deceased). Although some might seek to argue that women who are exposed to violence are justified in fatal assaults on their perpetrator, it should be remembered that for many years, men who killed their wives would seek to justify their actions on the grounds that they were being “nagged” or belittled by their wives. This kind of defensive strategy was condemned by feminist lawyers on the grounds that it rested on gender role stereotypes about women being “nags”; but one might argue that gender role stereotypes include narratives about men always being coercive and controlling and women always being victims.

The recent media attention on the case of a British woman, Penelope Jackson, provides an example of this phenomenon. Mrs. Jackson killed her husband, then called the ambulance and police to say that she had done so. Her crime was statistically highly unusual, given that she was a woman, in her seventh decade, with no prior record of violence or criminality and no other risk factors for violence. At trial she presented herself as a victim of coercive control, but the jury did not accept this and she was convicted of murder. Exposure to trauma was offered as an explanation for her violence, which made her seem more “normal” as a woman, and may have mitigated the sentence that she would receive.

Risk factors for violence by women appear similar to those for men: a history of delinquency in childhood, substance misuse and intergenerational transmision of violence (76). Psychological formulations of violence are crucial to the process of violence risk assessment, which is a key professional activity for forensic psychiatrists and psychologists in prisons and secure hospitals. However, violence risk assessments that are frequently used to assess individual risk profile are usually validated in males, making their use in female prisoners and patients questionable (77). Such violence risk assessments typically also rely on functional links between mental disorder and violence, and be based on samples of mentally ill violence perpetrators. These tools may therefore overlook relational components to violence, which is commoner in women (78).

## Caring for Women in Forensic Secure Units and Prisons

The criminal justice system has been criticised for neglecting the specific needs of women; the (79) Corston Report highlighted that “women have been marginalised within a system largely designed by men for men.” This is concerning, as numbers of female prisoners are rising globally; approximately 105,000 more women are in prison today compared to ten years ago (80, 144). This trend is of importance to forensic mental health practitioners, as incarcerated females are more likely than both the general population and male prisoners to suffer from mental health problems, engage in self-harming behaviour and commit suicide (81, 82). Despite this, men are still consistently more likely to be admitted to secure inpatient settings than women (83, 84).

When women are convicted of violent crime, they will be detained in prisons or secure psychiatric units, just as men are. However, in general female violence perpetrators are seen as lower risk than their male counterparts, and the female prison estate is far smaller than the men’s. In terms of secure psychiatric care, there are less than 10 high secure beds for women in England and Wales (compared 700 for males). Most female forensic patients are cared for medium or low secure services. In the United Kingdom, only 10% of patients detained under restriction orders are female (these are orders reserved for individuals deemed to pose a high risk of harm to others); and the proportion of women is decreasing, despite the numbers of restriction orders increasing between 2003 and 2016 (85).

There has been considerable debate about how best to provide gender sensitive care in forensic settings (86, 87). There seems to be some consensus that care and treatment needs to be segregated by sex (88); and services that have seen mixed sex services have also had reports of boundary violations between staff and patients, abuse of female patients by male patients and a lack of dignity for females in secure care (89).

Concern about the approach to female forensic care led to the United Kingdom’s Department of Health releasing new guidance in 2002 and 2003 (90, 91), which invited services for women to focus on women’s experience of trauma and on relational security ((92). This is in contrast to male services which emphasise enduring risk of violence and physical security. There remain concerns that female services still use models of care designed for male offenders and only later adapted, with little information about the necessity and value of any gender-based adaptations. (93, 94).

There has been some study of the value of gender-sensitive approaches in forensic services, mainly within correctional settings (95). Most of these gender sensitive approaches involve (a) increased attention to trauma in the lives of female prisoners and (b) increased availability of therapy. For example, Walker et al. (94) demonstrated the benefits of psychodynamic interpersonal therapy in women’s prisons in England in reducing self-harm, and an offender personality disorder (OPD) strategy for women has also been developed, bringing mental health professionals together with probation workers to provide psychologically informed treatment and risk management (93). Dedicated facilities in Australia have been developed for women with complex psychological issues (96) and Zielinski et al. (97) describe group therapy as an effective intervention for incarcerated women who have experienced sexual victimisation. In secure hospitals, psychological treatment programmes for women with dual diagnoses have also been introduced (98). Services need to make special provision for detained women (whether in prison or secure care) who are mothers and/or pregnant at the time of detention. Friedman et al. (99) highlight that perinatal mental illness rates are likely to be higher in prison and that pregnancy, lactation and menopause all affect prescribing choices in complex ways. Some sex-specific needs are undeniable; in the United States, around 4% of women enter prisons pregnant, and most are of child-bearing age (100).

Across the international literature, there appears to be an emphasis on understanding the experience of trauma in the lives of violent women, and its relevance for planning treatment and care (101, 102). Such a trauma-informed approach is seen as gender sensitive, yet as mentioned previously, levels of childhood adversity are similar in both male and female prisoners. Exposure to trauma in childhood is a risk factor for violence for both sexes; especially physical child abuse and witnessing domestic violence by carers, neither of which are specific to female children. De Vogel et al. (103) noted that women in forensic services were severely traumatised and had more complex histories of victimisation than men; but it is possible that women feel more able to discuss these histories than men do, and it is possible that men are not even asked about childhood trauma nor adult trauma because of gender role stereotypes (104). The relationship between childhood trauma and later violent offending is complex, and may be mediated by post-traumatic disorders but it is not confined to women (105, 106).

Overall, as Tolland et al. (107) highlight, there is a lack of available literature reviewing the value of gender specific interventions; and (it might be added) what the purpose of these interventions are for the women in custody. Given that the women are detained for having posed a serious risk of harm to others, it would make sense to try and demonstrate that both gender specific and trauma-informed interventions make some contribution to the formulation that links mental illness trauma exposure and violence. Of course, incarcerated women want compassion and better access to health care (108); but will this also help them reduce their risk of cruelty to others? Does providing trauma treatment improve later violence risk? If so, then males also need this intervention, and the more that is provided, the better the cost-offset benefits will be in terms of length of stay and detention. Trauma-informed therapies have been shown to work for men and women (109), suggesting that perhaps the genders are indeed more alike than different (13).

Why female forensic services should be trauma informed but not male services is puzzling; and would seem to reflect a kind of bias toward presenting violent women as victims not perpetrators. However, given that most victimised women do not perpetrate violence, the functional link between trauma exposure and violence risk will be complex to formulate. This is crucial for detained women, especially those in prison who will have to demonstrate reduction of risk to others before they can be released. If a female prisoner has done no psychological “work” on her offence and her capacity for cruelty, then she is unlikely to present successfully at a parole hearing. There are a large number of women in prison who are not able to access therapeutic interventions that address their violence and cruelty to others; is especially those who have killed family, partners or children.

Women may be detained in prisons and secure settings for long periods because of ambivalence about how to assess their risk (107). Women stay longer in medium and high secure units in England and are more frequently re-admitted compared to men (89). Exaggeration of risk may arise because of the rarity of female violence (especially if the crime has a high public profile and is disturbing) or because detained women may show high levels of disturbed behaviour (107). Detained women often use verbal abuse against staff, which takes a heavy emotional toll on professionals; and is viewed as far greater than working with men by forensic clinicians (92, 110). Three Canadian studies found that women are more frequently secluded than men (111–113), which may suggest either professional anger or helplessness with women who are perceived as “difficult” or threatening. In a Swedish study, (114) noted that when women in forensic care deviated from feminine gender norms, efforts were made to “normalise” their behaviour in order for them to become “acceptable.”

In summary, the needs of violent women resemble those for violent men in terms of common risk factors, especially previous mental health issues, early childhood adversity and substance misuse. However, there may important differences in terms of the level of physical violence inflicted on others and women’s apparently increased willingness to direct violence to their own bodies in the form of self-harming behaviour. These differences may also be influenced by gender role stereotypes in the women themselves as well as the criminal justice systems (115, 116).

Parkes and Freshwater (69) rightly point out the dangers of caring for women in forensic settings, from becoming embroiled in gang mentalities (117), being on the receiving end of demeaning attitudes of staff (118) and the risk of becoming re-traumatised in secure care (119). However, these are equally as likely consequences for men in similar circumstances.

## Legal Responses to Female Violence and Gender Role Stereotypes

Violent women violate gender role stereotypes because (a) they are unusual as perpetrators and (b) unusual compared to non-violent women. There has been some exploration of how the criminal justice system may support stereotypes of women as essentially passive by depicting violent women as mentally ill, vulnerable or coerced in some way. For example, in a Canadian study, women were twice as likely to be found unfit to stand trial following a violent crime than men, even after controlling for age, psychosis, forensic history and offence severity (120). They are also more likely to be declared mentally ill and diverted to hospital for treatment following homicide and subsequent legal insanity evaluations (121). Wilson et al. (122) found that forensic experts were more likely to mention and explore substance use issues for men, and stress and relationship problems with women. There is some anecdotal evidence that when women are charged with violence alongside male co-defendants, the defence strategy will argue that the women were coerced by antisocial partners into committing acts of violence as if they were passive participants who lacked autonomy. Carlyle et al. (123) showed that media outlets portrayed female IPV perpetrators as emotional with a history of abuse, and needing assistance from male accomplices when carrying out violent acts.

Women who abuse and assault children are often presented as “monsters,” and the defence will seek to normalise them in the criminal court. Wilczynski (74) has argued that mental illness is used to explain women’s violence to children in legal settings because this “explanation” not only reduces legal culpability and public condemnation but also to enable female violence perpetrators to fit better into the gender role stereotype of a “normally mentally unwell” woman. Another example of this may be found in the “offence/defence” of infanticide, used in cases of infant deaths at the hands of a woman when the “balance of her mind was disturbed”; feminists have criticised this as medicalising offenders and ignoring wider societal causes for such crimes (124, 125). This phenomenon may also extend to sentencing outcomes. Women who have sexually offended tended to receive more lenient sentences than their male counterparts (126). This suggests a denial of female violence in the court, as well as a reluctance to understand or accept female violence outside of mental illness (127).

Violent and cruel women seem to attract more social condemnation than their otherwise similar male counterparts (128). Women convicted of offences involving violence and cruelty attract excessive and emotional attention from social and press media and responses appear to be polarised. As described above, if children are involved, women may be more likely to be seen as “monsters”; they may also be held to blame not only for their own actions, but for the actions of their male partners, if those partners are not available to be publicly condemned (for example, Rose West and Ghislaine Maxwell).

## Gender Role Stereotypes and Women Working as Professionals in Forensic Settings

We conclude with some discussion about how gender role stereotypes might influence the work of women who work as professionals in forensic domains. Female forensic psychiatrists have long operated within traditionally male-predominant systems: namely those of medicine, law and the criminal justice system (129). Numbers of senior female clinicians are rising, but men still outnumber women in this field (130). The same gender imbalance seen in the forensic patient cohort is mirrored in forensic psychiatrists; in the United Kingdom 38% of forensic psychiatrists are women, compared to 25% in the United States (130, 131).

Most forensic services involve the control of male violence perpetrators by male custodians, and female professionals are still a minority. They may feel under pressure to behave like their male counterparts, and fear being perceived as “soft” in terms of discipline or boundaries in secure psychiatric settings. They may be encouraged to work with female offenders, as if they had something in common with them or could understand them better; and they may be assumed to be more at risk than their male colleagues of being attacked or offended by patient behaviours. Mercer and Perkins (132) explored female staff experiences of working with sexual offenders. Female nurses reported that they became absorbed in a stereotyped discourse in a “male” institutional space that assigned them sexual identities as opposed to professional. Therapeutic work related to sexual offending risk was deemed as “a job for the boys,” who also provided “safety” and security’ within the unit. The authors concluded that in this environment, female staff constructed themselves as “both at risk and inviting risk,” as a product of their gender.

The challenges of being female in such masculine environments are multiple. Forensic units are largely comprised of male patients with antisocial tendencies, many of whom will have had traumatic childhoods and dysfunctional or abusive relationships with their primary attachment figures, usually their mothers. The power imbalance between female clinician and male patient is especially obvious in such a setting, and may give rise to a host of difficult emotions, from humiliation to rage and perhaps sexual arousal.

Crewe (133) reported that incarcerated men may sexualise female staff presence, objectifying them and undermining their professional authority. There is evidence that female forensic workers are more likely to enter into boundary-violating sexual relationships with their patients (134). There is a complexity here which is that females in forensic settings are arguably in powerful “male” roles, and their patients in “female” roles in terms of passivity; but male forensic patients are often detained in secure settings because of their capacity for manipulation and deceit (135). Theodorou and Ali (136) highlight the dangers of reducing sexual boundary violations by female professionals into female “victim” and male “perpetrator” roles. However, in terms of professional ethics, the female professional has “abused” their male victim, and their hostility to their patients may be sexualised *precisely because* of the power discrepancy which is denied.

Gender roles may also influence the type of work that professionals engage with; the role of expert witness in the court is a traditionally male one, from which women have been historically excluded (137). In the United States, it is reported that female forensic psychiatrists are less likely to undertake work as an independent expert in the criminal court. Here, female forensic psychiatrists are also twice as likely to believe that gender is a factor in the selection of forensic experts in the court when compared to their male counterparts (130). In a commentary on the study by Price et al., Hackett (138) identified potential “hassle factors” as a possible explanation for this perception. She suggested that such factors might include subtle disrespect toward the expert (e.g., failing to provide information) and unrealistic last-minute time demands, and recommended that these be explored.

For those women that do become involved in such work, Ednie (129) highlighted different communication styles for men and women, as women used more indirect and less arrogant styles, which in turn are linked to lower credibility. Daftary-Kapur et al. (139) found that female experts were more likely to be recipients of intrusive questioning. Overall, jurors rate male experts as more likeable, believable, trustworthy and confident (140). Although Kaempf et al. (141) did not find a significant effect of expert witness gender, they did find that females were more likely to report being improperly addressed in the court, suggesting that subtle differences in attitudes are present. Some studies found some advantages of being female experts, e.g., within family court settings or in cases of battered women (137, 142).

The literature on the impact of gender role stereotypes and expert evidence is largely American and it is not possible to say with confidence that the same patterns exist in other parts of the world. The reasons for this may be influenced by social role theory and normative gender expectations: men are generally expected to be more controlling, assertive and independent than women, traits that are favourable when undertaking work in the criminal justice system (143). It is clear that there are gender differences at play in this domain, and further exploration of gender role stereotypes is needed internationally, especially within inquisitorial systems.

## Discussion

We have set out here some evidence to support a claim that that gender role stereotypes may be active and influential in forensic psychiatry: in terms of how violence is formulated, which diagnoses are made in violence perpetrators, how violent women are “seen” differently to men and how the law may treat violent women differently. We do not claim that this brief paper is definitive: the study of gender role stereotypes is vast and we have touched on only a few aspects here. We are limited by data mostly from western societies. However, we argue that there is enough existing evidence to suggest that gender role stereotypes may be operating in subtle or not so subtle ways in the forensic domain, and may affect how both male and female violence perpetrators are seen by professionals, and how female professionals are seen by others.

Violence is not usual human behaviour; and violent women are unusual people. It can therefore be hard to establish evidence in this field, especially given that forensic services are a minority of mental health provision. We have a particular concern that failure to take women’s cruelty seriously may lead to them being deprived of the kind of interventions that might help them desist from future cruelty. We suggest that focussing only on female violence perpetrators as traumatised, without paying due attention to their perpetrator experience, may be especially disabling for women; and deny them agency over their future risk in ways that are not the case for their male counterparts. Conversely, despite similar rates of childhood adversity in both genders, we are concerned that male offenders are not receiving trauma-based interventions that might make a difference to both mental health and future risk.

Gender role stereotypes are also perpetuated by the international media. Extensive attention is paid to violent women, particularly women who kill, inflict cruelty on children or who are involved in sexualised offences. Women appear to be typecast into roles of coerced victims, or accomplices to male partners. Alternatively, they are portrayed as monsters beyond retribution, as they have violated their expected roles as wives, mothers and partners.

Within the profession, there are more women than before; but still less than in other branches of medicine and psychiatry. There is a paucity of evidence examining gender bias within forensic settings. There does appear to be some evidence that female expert witnesses are viewed differently, and less favourably, to males in court settings. We wonder if forensic psychiatry is still seen as a largely male subspeciality, and if so, whether gender role stereotypes influence this. There is also some evidence to suggest that gender bias exists as part of daily life for female forensic professionals working in secure settings, prohibiting them from carrying out particular therapeutic tasks and typecasting them into dependent roles.

Overall, we suggest that it is time for the training of forensic clinicians to include close attention to gender role stereotypes and how they might consciously or unconsciously affect formulations of violence and its management. There may be ethical aspects to consider, especially if the influence of gender role stereotypes leads to offenders and professionals being treated unfairly and unjustly. Additionally, we recommend further research into the experience of both female patients and offenders, as well as female mental health professionals, who are navigating systems designed by men, for men.

In summary, it is disparities of power and vulnerability that have traditionally driven discourses of sex and gender; disparities that also exist within the field of forensic psychiatry. This paper is an invitation to increase awareness of gender as a social construct, which may be operating in forensic settings. If we do not explore and address these issues, there is a risk that forensic services will parallel the societies that caused such damage to our patients, and patient care will be affected in ways that are harmful.

## Author Contributions

SA and GA discussed the scope of the manuscript and jointly contributed to the final manuscript. Both authors contributed to the article and approved the submitted version.

## Conflict of Interest

The authors declare that the research was conducted in the absence of any commercial or financial relationships that could be construed as a potential conflict of interest.

## Publisher’s Note

All claims expressed in this article are solely those of the authors and do not necessarily represent those of their affiliated organizations, or those of the publisher, the editors and the reviewers. Any product that may be evaluated in this article, or claim that may be made by its manufacturer, is not guaranteed or endorsed by the publisher.
